# S100A8 & S100A9: Alarmin mediated inflammation in tendinopathy

**DOI:** 10.1038/s41598-018-37684-3

**Published:** 2019-02-06

**Authors:** Lindsay A. N. Crowe, Michael McLean, Susan M. Kitson, Emma Garcia Melchor, Katharina Patommel, Hai Man Cao, James H. Reilly, William J. Leach, Brain P. Rooney, Simon J. Spencer, Michael Mullen, Max Chambers, George A. C. Murrell, Iain B. McInnes, Moeed Akbar, Neal L. Millar

**Affiliations:** 1 Institute of Infection, Immunity and Inflammation, College of Medicine, Veterinary and Life Sciences University of Glasgow, Glasgow, Scotland UK; 2 Department of Orthopaedic Surgery, Queen Elizabeth University Hospital Glasgow, Glasgow, Scotland UK; 3 Orthopaedic Research Institute, Department of Orthopaedic Surgery, St George Hospital Campus, University of New South Wales, New South Wales, Australia

## Abstract

Alarmins S100A8 and S100A9 are endogenous molecules released in response to environmental triggers and cellular damage. They are constitutively expressed in immune cells such as monocytes and neutrophils and their expression is upregulated under inflammatory conditions. The molecular mechanisms that regulate inflammatory pathways in tendinopathy are largely unknown therefore identifying early immune effectors is essential to understanding the pathology. Based on our previous investigations highlighting tendinopathy as an alarmin mediated pathology we sought evidence of S100A8 & A9 expression in a human model of tendinopathy and thereafter, to explore mechanisms whereby S100 proteins may regulate release of inflammatory mediators and matrix synthesis in human tenocytes. Immunohistochemistry and quantitative RT-PCR showed S100A8 & A9 expression was significantly upregulated in tendinopathic tissue compared with control. Furthermore, treating primary human tenocytes with exogenous S100A8 & A9 significantly increased protein release of IL-6, IL-8, CCL2, CCL20 and CXCL10; however, no alterations in genes associated with matrix remodelling were observed at a transcript level. We propose S100A8 & A9 participate in early pathology by modulating the stromal microenvironment and influencing the inflammatory profile observed in tendinopathy. S100A8 and S100A9 may participate in a positive feedback mechanism involving enhanced leukocyte recruitment and release of pro-inflammatory cytokines from tenocytes that perpetuates the inflammatory response within the tendon in the early stages of disease.

## Introduction

Overuse injuries of the tendon—encompassed by the term ‘tendinopathy’—represent a largely underestimated group of musculoskeletal disorders associated with chronic inflammation and dysregulated tissue repair^1^. Tendinopathies account for 30–50% of all sporting injuries and a high proportion of rheumatological and orthopaedic referrals from primary care physicians^2^. Despite historical disagreement between ‘inflammation’ vs ‘degeneration’ hypotheses it is now widely accepted that inflammatory mechanisms elicited by persistent mechanical injury at a microscopic level disturb the intricate homeostatic balance that exists between stromal and immune cell compartments within the tendon during the initial stages of disease^3,4^.

Expression of inflammatory mediators and associated immune cell infiltration is most pronounced in the early stages of tendinopathy^5^. Several experimental models have implicated cytokines as early immune effectors in stromal pathologies and essential mediators of tendon repair. Expression of cytokines and chemokines including IL-1, IL-33, IL-10, CCL2 and CXCL12 have been demonstrated in tenocytes and blockade of tumour necrosis factor alpha (TNF-α) was shown to improve the mechanical strength of tendon-bone healing in a rat rotator cuff repair model^6–9^. Conversely, surgically injured tendons of IL-6 deficient mice show reduced healing and inferior mechanical properties compared to normal injured controls^10^. In a rat model of Achilles tendon injury increased infiltration and accumulation of immune cells including neutrophils and macrophages was observed between 1 and 28 days post injury^11^. Subsequent human studies have identified distinct populations of myeloid monocytes and macrophages in both early and late tendinopathy^5,12^.

Monocytes recruited to areas of damage enter tissue in response to activation of chemokine pathways such as the CCL2/CCR2 axis^13^. In addition to monocytes, the myeloid compartment within the tendon may also include mature tissue macrophages that are programmed to respond to chemotactic factors following injury and assist in the initial inflammatory response. Current evidence suggests that immune cell infiltration and inflammatory mediators play diverse roles in the initiation and maintenance of tissue repair^14^. In the context of tendinopathy an initial inflammatory response promotes beneficial healing; however, sustained inflammatory conditions may eventually lead to dysregulated matrix remodelling.

Mobilization of immune cells within the tendon matrix is likely precipitated by microenvironmental changes that occur in response to injury^15^. Alarmins, also referred to as damage associated molecular patterns (DAMPS), are endogenous molecules rapidly released into the extracellular milieu following tissue damage^16^. S100A8 and S100A9, also known as myeloid related protein 8 (MRP8) and MRP14, are low molecular weight calcium binding proteins constitutively expressed by cells of myeloid origin^17^. Under pathological conditions they are induced in other cell types in response to environmental triggers. Acting as alarmins they are released passively by necrotic cells or by active secretion from activated immune cells^18^. Extracellular S100A8 and S100A9 bind pattern recognition receptors (PRRs) including Toll-like receptors (TLRs) and receptor for advanced glycation end products (RAGE) to activate the innate immune system and mediate inflammation by influencing monocyte and macrophage behavior^19^.

Both S100A8 and S100A9 are chemotactic for monocytes and have been implicated in myeloid cell maturation where their expression directly correlates with state of differentiation^20,21^. Moreover, they may exert both pro and anti-inflammatory effects by manipulating the cytokine profile of cells through PRR binding^18^. S100A8 and S100A9 are considered biomarkers of disease activity in chronic inflammatory pathologies associated with impaired matrix remodeling such as rheumatoid arthritis (RA), inflammatory bowel disease (IBD) and cystic fibrosis^22^. In addition, gene expression studies have documented the presence of S100 proteins in tendinopathy^23^. Recent investigations have established tendinopathy as an alarmin-mediated pathology^8,9^ thus we sought to characterize the expression of S100A8 and S100A9 in human tendinopathy and explore their biological significance in the context of inflammation and matrix regulation.

## Results

### S100A8 and S100A9 expression is increased in tendinopathy

**Figure 1:**
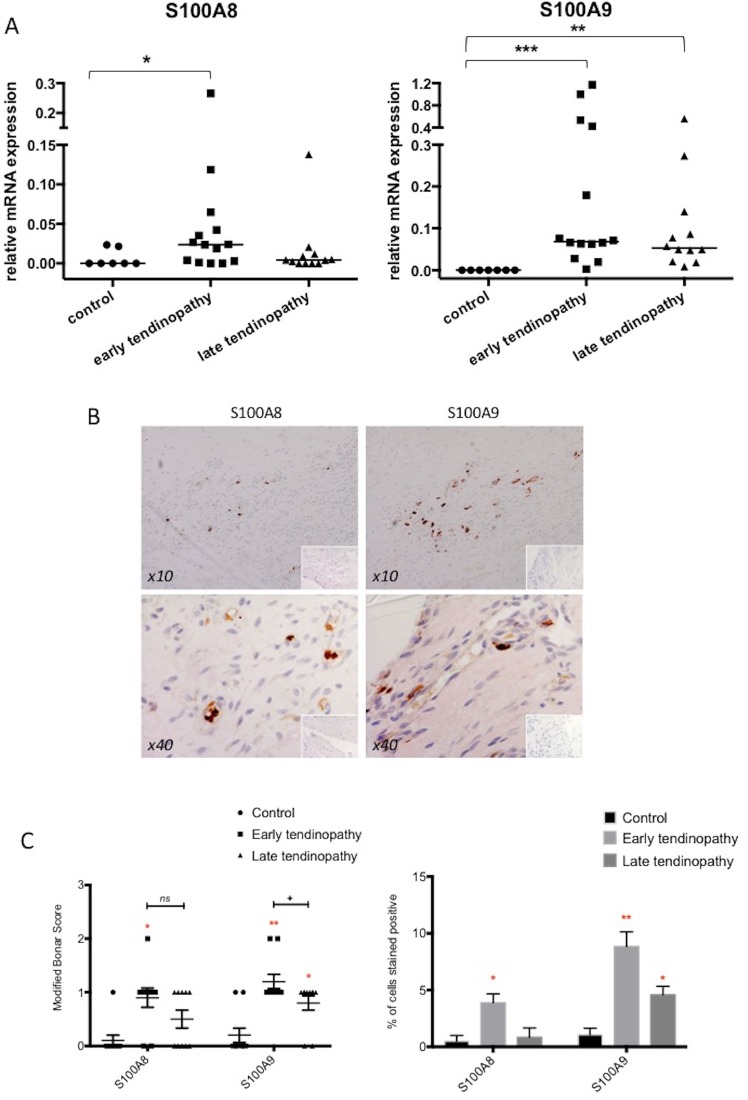
S100A8 and S100A9 expression is increased in tendinopathy. (**A**) Relative mRNA expression (2^-ΔCT^) of S100A8 & S100A9 in control (hamstring tendon, n = 7), early tendinopathy (intact subscapularis biopsy, n = 14) and late tendinopathy (torn supraspinatus tendon, n = 12). Data represent mean ± SEM relative to housekeeping gene GAPDH (mean of duplicate analysis). *p < 0.05, **p < 0.01, ***p < 0.001 (Kruskal-Wallis test) versus control. (**B**) Immunostaining of S100A8 and S100A9 in early tendinopathy (subsapularis biopsy) at 10x and 40x magnification. (**C**) Graphs illustrate modified Bonar scoring and percentage of cells stained positive for samples of human tendon biopsies for expression of S100A8 and A9 with mean and SEM shown. n = 10 for control tendon, n = 10 for early and late tendinopathy. Modified Bonar scoring system depicts mean score per sample based on five high-power fields. 0 = no staining, 1 = < 10%, 2 = 10%–20%, 3 = > 20% positive staining of cells per high-power field. *p < 0.05, **p < 0.01 (ANOVA) versus control biopsies. ^+^p < 0.05 late versus early tendinopathy.

S100A8 mRNA expression is significantly upregulated in early tendinopathy compared with control (p < 0.05) (Fig. 1A). S100A9 expression was more profoundly increased in both intact (p < 0.001) and torn tendon biopsies (p < 0.01) compared with control (Fig. 1B). We observed greatest significant upregulation of S100A8 and S100A9 in early tendinopathy and a relative absence of S100A8 mRNA expression in late tendinopathy suggesting these alarmins are key regulators in the early stage of disease. We next noted significantly greater protein expression of the alarmin molecules S100A8 and S100A9 in the early tendinopathy group compared with the control group (p < 0.05) (Fig. 1B). Both S100 proteins appeared not be localised around the stroma and sub analysis using back-to-back staining with the macrophage marker CD68 revealed that both S100A8 and S100A9 were localised to macrophage cells (Supplementary Fig. 1). Semi quantitative analysis again suggested that S100A9 (9% early tendinopathy, 5% late tendinopathy, % of cells stained positive) was more frequently expressed that S100A8 (4% early tendinopathy, 1% late tendinopathy, % of cells stained positive) in tissue biospies.

There were no significant correlations between S100A8 & A9 expression and the mean duration of symptoms, patient age or number of steroid injections (data not shown). Late tendinopathy samples exhibited marked degeneration, mucoid change and frank chondroid metaplasia (grade 4), whereas matched subscapularis tendon biopsies had grade 2–3 changes indicative of early tendinopathy. All control samples were classified as grade 1 consistent with normal fibrotendinous tissue with large distinct collagen fibrils.

### Mechanical injury stimulates release of CCL2 *in vitro*

**Figure 2:**
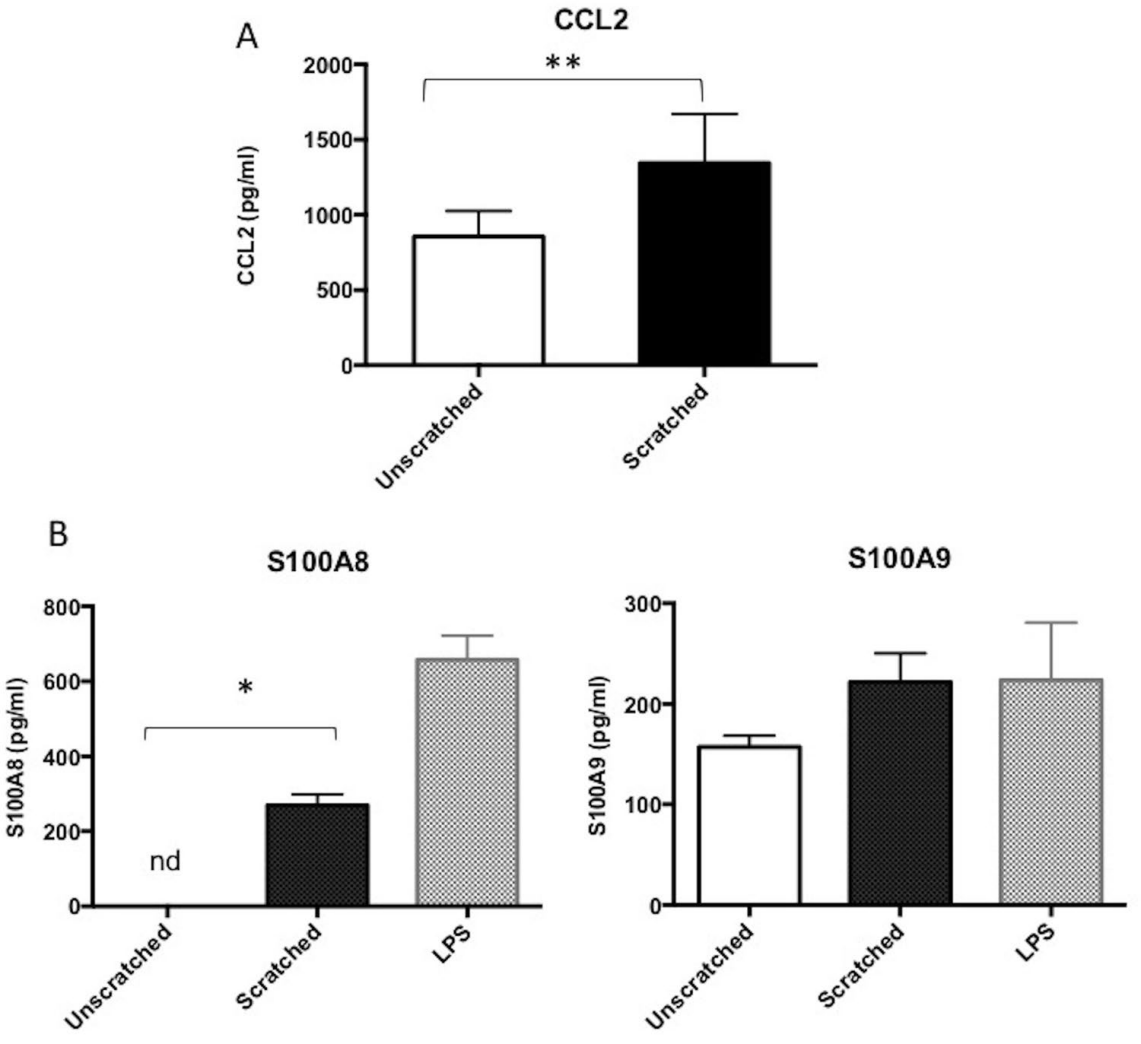
Damage induces release of CCL2 from tenocytes. (**A**) CCL2 release measured by ELISA in supernatants collected from tenocytes scratched and incubated for 24 hours post injury compared to unscratched control. (**B**) S100A8 and S100A9 concentration in supernatants from CD14^+^ monocytes (isolated from buffy coats) cultured in conditioned medium from tenocyte scratch assay for 24 hours. ‘nd’ indicates not detected. All data represent mean ± SEM. n = 3 donors *p < 0.05, **p < 0.01 versus control (Student’s *t*-test).

Given that alarmins are rapidly released into the extracellular compartment following tissue damage we sought to confirm the mechanism of release of S100A8 and S100A9 using an *in vitro* model of microtrauma and explore the potential inflammatory reaction of tenocytes after injury. Scratched tenocytes exhibit significantly increased release of CCL2 versus control (Fig. 2A, p < 0.01); however, we did not detect any induction of either CCL20 or CXCL10 release following injury indicating CCL2 release from tenocytes is the primary mechanism of immune cell mobilization following tendon injury.

As CCL2 is a known chemokine for monocyte recruitment we hypothesised that these cells are recruited post-injury within the tendon. To simulate the stromal environment following injury CD14^+^ monocytes were incubated with conditioned medium obtained from scratched tenocytes. We observed a significant increase in S100A8 release compared with unscratched control (p < 0.05) (Fig. 2B). Although not statistically significant, S100A9 release following incubation with tenocyte conditioned medium was also greater than unscratched and comparable to values observed with LPS stimulation (Fig. 2C).

### S100A8 and S100A9 do not directly alter matrix proteins *in vitro*

**Figure 3:**
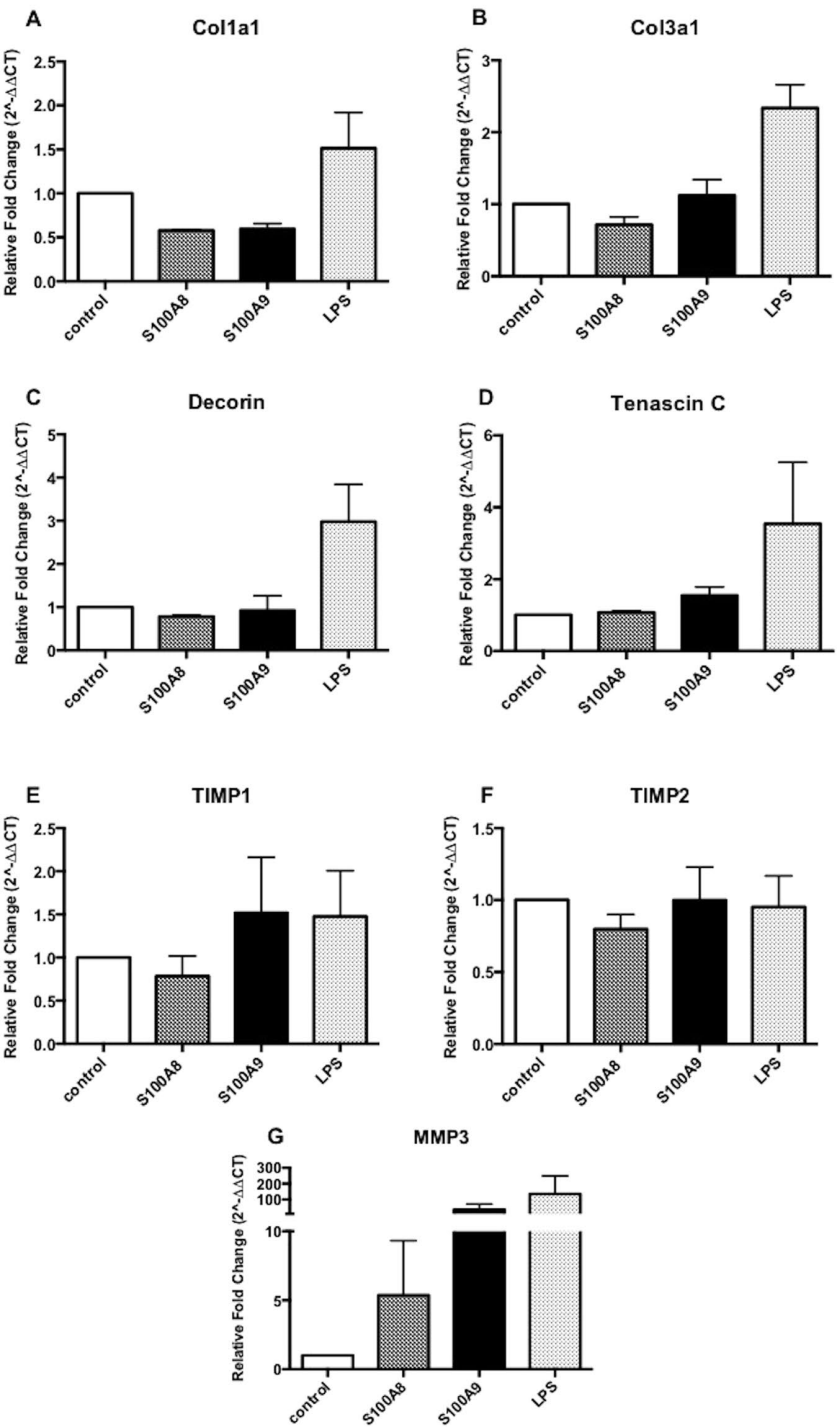
S100A8 and S100A9 do not directly alter matrix proteins *in vitro* (**A**) Col1a1, (**B**) Col3a1, (**C**) Decorin, (**D**) Tenascin C, (**E**) TIMP1, (**F**) TIMP2 and (**G**) MMP3 mRNA expression in tenocytes stimulated 1 µg/ml recombinant S100A8, 1 µg/ml recombinant S100A9 or 1 ng/ml LPS for 24 hours was determined by real time PCR. Data represent mean ± SEM of duplicate samples expressed as relative fold change normalised to control (unstimulated) samples, n = 3.

Previous investigations have shown that alarmins differentially regulate collagen synthesis and expression of matrix proteins in tendinopathy^8,9^ thus we sought to assess the effect of extracellular S100A8 and S100A9 on matrix regulation in primary human tenocytes. Neither S100A8 or S100A9 had any significant effect on Col1a1, Col3a1, Decorin or Tenascin C gene expression following 24 hours stimulation relative to controls (Fig. 3A–F).

To assess the effect of S100A8 & A9 on protease release in tenocytes we performed an array that allows simultaneous detection of 35 proteases. We found a marked increase in MMP3 expression between control vs S100A9 (Fig. S2). This was validated at protein level where a significant increase in MMP3 release from tenocytes was observed upon stimulation with S100A9 (Fig. S2A). Additionally, we found upregulation of MMP3 transcript in response to both S100A8 and S100A9 stimulation (Fig. 3G), however no increase in TIMPs 1 or 2 were observed. Furthermore, no effect was observed on collagen I protein release from tenocytes in response to S100 stimulation (Supplementary Fig. S3).

### S100A8 and S100A9 induce secretion of inflammatory mediators from human tenocytes

**Figure 4:**
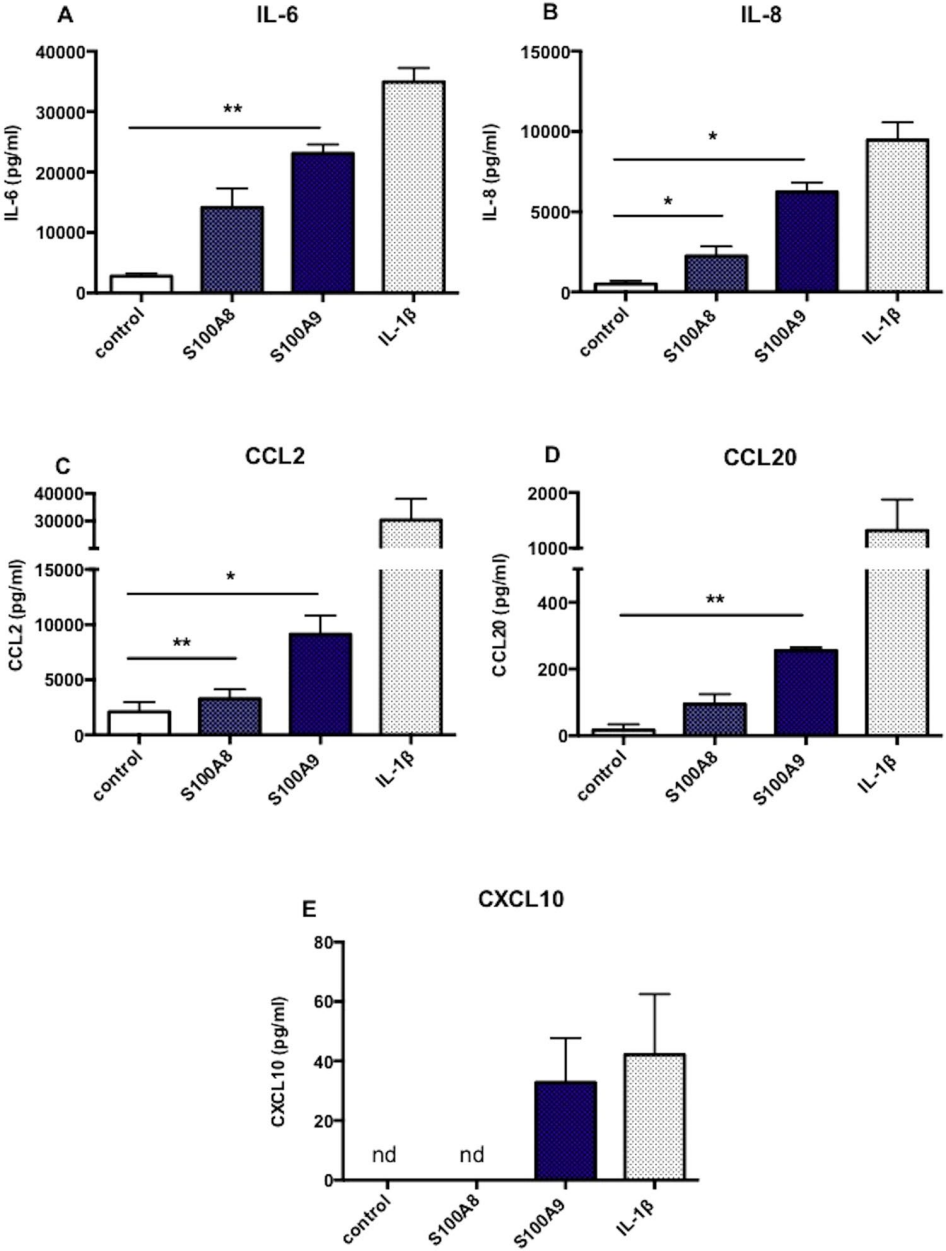
S100A8 and S100A9 induce cytokine and chemokine release from human tenocytes Tenocytes stimulated with 1ug/ml S100A8, 1ug/ml S100A9 or 10 ng/ml IL-1*β*. Concentrations of IL-6 (**A**) IL-8 (**B**) CCL2 (**C**) CCL20 (**D**) or CXCL10 (**E**) in cell culture supernatants measured following 24 hours stimulation. Values expressed as pg/ml. All data represent mean ± SEM, n = 3, *p < 0.05, **p < 0.01, ***p < 0.001 compared to control (unstimulated) samples (Student’s *t*-test).

We next explored the extent to which S100 proteins may influence the inflammatory microenvironment within the tendon post-injury. Recombinant S100A9 at a concentration of 1ug/ml significantly increased release of IL-6 (p < 0.01), IL-8 (p < 0.05) and CCL20 (p < 0.01) from tenocytes (Fig. 4). Furthermore, S100A9 stimulation induced a 40 fold increase in CXCL10 expression compared to control (not detected). S100A8 stimulation displayed the same trend of significant IL-8 (p < 0.05) and CCL2 (p < 0.001) release from tenocytes; however, increases in IL-6 and CCL20 release were not significant following 24 hours’ stimulation. Taken together, these data suggest S100A8 and S100A9 play a role in the activation of resident tenocytes and initiate a cascade of inflammatory processes.

## Discussion

Our study has established the presence of S100A8 and S100A9 in a human model of tendinopathy, most notably in the early stages of disease. Coupled with confirmation of passive release of CCL2 in response to acute injury our data supports the concept of S100A8 & A9 acting as functional tissue alarmins in tendinopathy by promoting immune cell recruitment. We have identified that S100A8 & A9 play an important immunomodulatory role in tendinopathy through activation of the innate immune system and manipulation of the stromal microenvironment.

Increasingly, alarmins are becoming recognized as key regulators in musculoskeletal pathologies^16,24^. S100A8 and S100A9 are considered surrogate markers of disease activity in RA where serum concentrations directly reflect levels of active inflammation^25,26^. In addition, S100A8 & A9 promote recruitment of inflammatory monocytes to the synovium in a murine model of osteoarthritis and blockade of S100 signalling ameliorates inflammatory processes^27^. Previous investigations in human models of tendinopathy have consistently established the presence of alarmins in diseased tendon and revealed functional roles *in vitro*^8,9,28^. Most recently we have demonstrated the alarmin HMGB1 regulates expression of inflammatory cytokines and matrix changes in tenocytes in a TLR4 dependent manner^9^. Observations taken from the present study indicate S100A8 and S100A9 regulate expression of inflammatory mediators *in vitro* which corroborates previous findings and suggests S100A8 & A9 are likely acting through DAMP receptors to influence downstream transcription and release of CCL2, CCL20, CXCL10, IL-6 and IL-8.

Hallmark features of tendinopathy include dysregulated collagen synthesis with a detrimental transition from type 1 to an inherently weaker type 3^29^. In addition, ECM turnover is regulated by non-structural matricellular proteins such as decorin and tenascin C that are thought to be upregulated under inflammatory conditions and in response to mechanical strain^30^. Interestingly, the present study did not find any changes in collagen, decorin or tenascin C in tenocytes in response to S100A8 & A9 stimulation. This may reflect differences in endogenous activity of various DAMPs. In addition to passive secretion from necrotic cells, under pathological conditions intracellular alarmins reserve the potential to be secreted from activated immune cells. In contrast to the relatively slow translocation dependent secretion of nuclear HMGB1^31^, active secretion of S100A8 & A9 from monocytes is rapid and energy dependent^32^ suggesting they are inherently programmed to facilitate early inflammatory responses rather than directly alter extracellular matrix production.

Protease screening confirmed induction of MMP3 in response to S100 stimulation. MMPs are generally considered to be fibrinolytic modulators of extracellular matrix turnover and have been associated with ongoing tissue damage and development of chronic disease^33,37^. Evidence suggests they are also involved in regulation of inflammatory processes including cytokine processing and activation^34,35^, and leukocyte migration^36^. We did not observe any changes in other matrix proteins or tissue inhibitors of metalloproteinases (TIMP1 and TIMP2) therefore, in the context of tendinopathy, MMP3 may regulate inflammation in addition to influencing matrix remodelling.

Recent evidence has revealed that stromal fibroblast activation markers are persistently upregulated in diseased tendon^38^. In response to recurrent injurious or inflammatory stimuli stromal cells are subject to phenotypic transformations that alter their functional properties; such adaptations reflect the activation state of the cell population. Typically, stromal cell activation is characterised by rapid induction of cytokines, chemokines and extracellular matrix components^39^. We observed an induction of CCL2, CCL20, CXCL10, IL-6 and IL-8 release from tenocytes in response to S100A8 & A9 stimulation suggesting their primary action may be activation of the resident tenocyte population. This, in turn, will promote immune cell recruitment and influence the nature of stromal microenvironmental cues.

The CCL2/CCR2 axis is primarily associated with the initial recruitment of classical inflammatory monocytes to sites of inflammation or tissue damage^40,41^. Within the tendon a portion of the recruited monocytes may continue development into macrophages following egress into the stromal microenvironment. It is thought classical monocytes recruited by CCL2 are programmed to differentiate more readily into inflammatory macrophages and may promote excessive inflammation^42^. Conversely, synergistic activities of IL-6 and CCL2 have been shown to induce alternative activation of myeloid monocytes^43^. As such, the recruitment and development of monocytes and macrophages is highly niche specific and undifferentiated monocytes/macrophages retain the potential to differentiate according to their environment. Given the complex nature of the stromal microenvironment is it likely that both ‘classical’ inflammatory macrophages and ‘alternatively activated’ macrophages exist as a dichotomy that drives a state of chronic inflammation.

**Figure 5:**
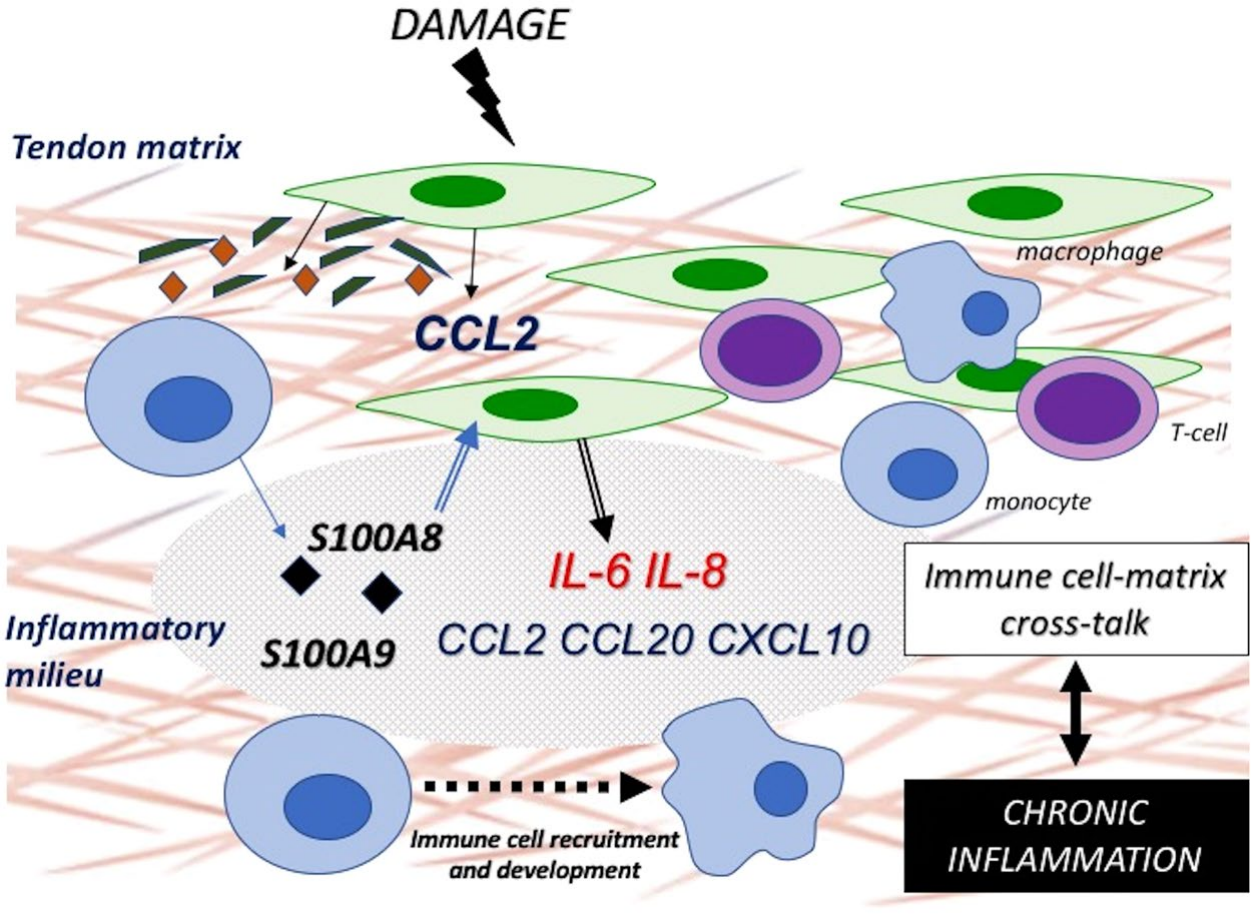
S100A8 and S100A9 promote immune cell recruitment and development in tendinopathy Proposed mechanism whereby damage induces release of CCL2 from tenocytes recruiting monocytes to the site of injury. Soluble factors stimulate release of S100A8 and S100A9 from monocytes recruited to the tendon matrix. S100A8 and S100A9 bind receptors on the tenocyte surface to induce release of cytokines and chemokines promoting further recruitment and development of immune cells. Formation of an inflammatory microenvironment facilitates immune cell-matrix cross talk and promotes a state of chronic inflammation.

Based on our observations using a previously explored *in vitro* injury model we propose that acute injury induces CCL2 mediated monocyte recruitment^44^. Here, soluble factors or biologically active ECM fragments induce subsequent release of S100A8 and S100A9 from monocytes that will, in turn, bind receptors on the tenocyte surface and stimulate release of further inflammatory factors (Fig. 5). Within the tendon matrix monocytes continue development to mature macrophages and differentiate according to environmental cues. Histologically the presence of mononuclear cells is associated with a state of chronic inflammation^45^. Excessive immune cell infiltration and the presence of macrophages may contribute to persistence of inflammation and promote immune cell-matrix crosstalk that drives inflammatory healing characterised by aberrant and inferior matrix repair. It may be postulated in the context of this study that CCL2, CCL20 and CXCL10 mediate initial inflammatory monocyte recruitment while IL-6 and IL-8 participate as potent inflammatory factors in the acute phase response. This will subsequently act to promote a transitory state towards established chronic inflammation. In addition to monocyte recruitment these chemokines are capable of recruiting T cells, mast cells and natural killer cells that will likely contribute to the development of a complex and dynamic inflammatory milieu.

A recent study detailing the expression of alarmins in the fibrotic disorder adhesive capsulitis demonstrated S100A8/S100A9 expression is localised to immune cells; specifically, CD68^+^ macrophages^46^. Our data describing S100A8 & A9 release from monocytes highlights the importance of activated immune cells as a source of alarmins in diseased tendon. Under inflammatory conditions S100A8 and S100A9 are known to regulate positive feedback mechanisms thus it is plausible tenocytes and activated monocytes/macrophages mutually amplify levels of extracellular alarmins and promote phagocyte recruitment^47,48^. Furthermore, S100A8/S100A9 are involved in myeloid cell differentiation and induce secretion of pro-inflammatory cytokines including TNF-α and IL-1ß from monocytes^49^; it is likely these mediators will also act to exaggerate and sustain inflammatory conditions.

There are limitations inherent in our study. Age-related changes within the tendon samples could contribute to the degenerative picture and alarmin expression seen in the matched subscapularis tendons. However, the lack of degenerative change on MRI and arthroscopic examinations suggests that the differences are truly at the cellular level as suggested by our work. Subscapularis tendon is functionally and organizationally distinct from supraspinatus thus responds to mechanical loading in a different manner that may alter its cellular profile. Control samples from subscapularis undergoing stabilisation may not be truly ‘normal’ controls but are currently the best available control tendon sample and this is reflected by a Bonar score of 1. It is reassuring, however, that we found the same inflammatory cell subtypes in matched subscapularis tissue indicating that the inflammatory response may be uniform within joints subjected to tendon degeneration. In addition, having subscapularis samples from the same patient eliminates bias that may result from variation between individuals and has been previously shown to be useful method in sampling of tissues. We also point out that, while our human tissue biopsies show the presence of S100A8 & A9 at mRNA and protein level, the majority of our mechanistic work utilises an early disease *in vitro* culture system. Further mechanistic investigation in an *in vivo* tendon model and late disease *in vitro* cultures would more likely address the hierarchal role of S100 molecules in tendon pathogenesis.

## Conclusions

Our data confirms the presence of S100A8 and S100A9 in tendinopathy and suggests they actively contribute to pathological proceedings in the early stages of disease. We propose that by modulating the stromal microenvironment S100A8 and S100A9 promote recruitment of inflammatory cells to the site of injury and support a detrimental transition from acute to chronic inflammation. Selectively targeting DAMP signalling in early disease provides scope for novel translational strategies in the management of tendon disorders.

## Materials and Methods

### Human model of tendinopathy

All procedures and protocols were approved by the Ethics Committee under approval numbers Central Network, South East Health (HREC/96/55, HREC/14/130) and West of Scotland REC (REC14/WS/1035) with informed consent obtained and carried out in accordance with standard operative procedures. For immunohistochemistry, ten supraspinatus tendon samples were collected from patients with rotator cuff tears undergoing shoulder surgery (Table 1). The mean age of the rotator cuff ruptured patients was 50 years (range, 30–62 years) —the mean tear size was 2.2 cm^2^ (range 1–4 cm^2^). Samples of the subscapularis tendon were also collected from the same patients. Patients were only included if there was no clinically detectable evidence of subscapularis tendinopathy on a preoperative MRI scan as determined by a musculoskeletal radiologist or macroscopic damage to the subscapularis tendon at the time of arthroscopy as determined by the senior author (NLM)—by these criteria they represented a preclinical cohort. In this cohort, all patients fulfilled the following criteria: (1) a history of shoulder pain and dysfunction, (2) no previous surgery on the affected shoulder, (3) no radiographic sign of fracture of the shoulder and (4) no history of RA or osteoarthritis^50^. An independent control group was obtained comprising ten samples of subscapularis tendon collected from patients undergoing arthroscopic surgery for shoulder stabilisation without rotator cuff tears, no previous shoulder surgery, no radiographic signs of shoulder fracture, or history of RA or OA. The absence of rotator cuff tears was confirmed by arthroscopic examination. The mean age of the control group was 23 years (range, 16–28 years). For mRNA expression, fourteen (mean 49 years range, 30–65 years) early tendinopathy (subscapularis) samples, 12 late (mean 54 years range, 38–68 years) tendinopathy (supraspinatus) samples were utilised while 8 hamstring tendons (mean 23 years (range, 15–25 years) obtained at the time of routine anterior cruciate ligament reconstruction were employed (utilised as control due to lack of subscapularis control RNA) as an independent control group. Additionally, standardised patient demographics were obtained preoperatively and included the duration of shoulder symptoms experienced by the patient and the number of subacromial steroid injections.

**Table 1:** Patient demographics.

Tear Size	Control	Small (<1 cm^2^)	Medium (>1–3 cm^2^)	Large (>3–5 cm^2^)
Number of cases	10	4	4	2
Mean age in years (range)	21 (16–28)	44 (30–58)	48 (46–62)	52 (42–53)
Mean duration of symptoms in months (range)	6.0 (2–12)	8.2 (2–18)	6.8 (2–16)	8.6 (5–18)
Mean number of steroid injections	0	1.8	1.1	1.7

### Cell culture and treatments

Human tendon-derived cells were explanted form hamstring tendons of patients undergoing anterior ligament reconstruction. Patients were age ranged from 18–25. Hamstring tendons were cut into 2 mm3 pieces and placed in RPMI 1640 supplemented with 10% heat-inactivated Fetal Bovine Serum (FBS), 100 µg/ml penicillin, 100 µg/ml streptomycin and 2nM L-glutamine (all Thermo Fisher Scientific). Explants were maintained in a humidified environment at 37 °C, 5% CO_2_ to allow tenocytes to adhere. Medium was replenished every 5 days until cells were confluent. Tissue was then removed and cells were placed in fresh medium. Cells were passaged using trypsin-EDTA (Sigma-Aldrich). Tenocytes from the second and third passage were seeded two days prior to stimulation.

Normal tenocytes were seeded at 2.5 × 10^4^/ml in 24 well culture plates and allowed to rest for 48 hours. Adherent tenocytes were stimulated with 1 µg/ml recombinant human S100A8 (Abcam), S100A9 (Abcam), 10 ng/ml LPS or 10 ng/ml IL-1ß for 24 hours in complete RPMI.

### Scratch assay

Normal tenocytes were seeded at 5 × 10^4^/ml in 12 well culture plates and scratched 4 times across the diameter of the plate with a sterile pipette tip. Injured cells were incubated for 24 hours before harvesting of supernatants^44,51^.

### Monocyte stimulation

Buffy coats from healthy volunteers were obtained from the Scottish National Blood Transfusion Service (SNBTS) under the reference 10–12 (v2). Human CD14^+^ monocytes were obtained from buffy coats by density gradient separation. Peripheral blood mononuclear cells were incubated with CD14 microbeads (Miltenyi) and posititvely selected via magnetic separation using autoMACS Pro Separator. CD14 + monocytes were seeded in 24 well culture plates at a density of 2.5 × 10^5^ per well with supernatants (diluted 1 in 2 with complete RPMI) obtained from the tenocyte scratch assay for 24 hours.

### RNA extraction, cDNA synthesis and real time qPCR

Tendon tissue was placed in RNA later solution (Ambion) and stored at −20 °C. Tissue samples were homogenized in PureLink lysis buffer containing 1% 2-mercaptoethanol using a Qiagen Tissue Lyser LT. Total RNA was isolated using the PureLink RNA Mini Kit (Thermo Fisher Scientific) according to manufacturer’s instructions.

Cells were placed in PureLink lysis buffer containing 1% 2-mercaptoethanol and RNA was extracted using mini columns according to the PureLink protocol.

RNA concentration and purity was determined using a spectrophotometer (Nanodrop 2000, Thermo Scientific). 100 ng of RNA was converted to cDNA using High Capacity cDNA reverse transcription kit (Thermo Fisher Scientific) according to manufacturer’s instructions. cDNA was diluted 1 in 5 using RNase free water. qPCR was performed using PowerUp Sybr Green Mastermix (Thermo Fisher Scientific) and 1 μl cDNA was used per reaction with 0.1 μM of forward and reverse primers. Each sample was run in duplicate and normalized to GAPDH or 18 S endogenous control. Data represents relative mRNA expression (2^−ΔCT^) or fold change from untreated cells (2^−ΔΔCT^).

Primers (Integrated DNA Technologies) were as follows:

***GAPDH*** (f) 5′-TCGACAGTCAGCCGCATCTTCTTT-3′ (r) 5′-ACCAAATCCGTTGACTCCGA CCTT-3′

***18S*** (f) 5′-GTAACCCGTTGAACCCCATT-3′ (r) 5′-CCATCCAATCGGTAGTAGCG-3′

***Col1a1*** (f) 5′-CAATGCTGCCCTTTCTGCTCC-3′ (r) 5′-CACTTGGGTGTTTGAGCATTG-3′

***Col3a1*** (f) 5′- TATCGAACACGCAAGGCTGTG-3′ (r) 5′-

GGCCAACGTCCACACCAAATT-3′

***S100A8*** (f) 5′-AGACCGAGACCGAGTGTCCTC-3′ (r) 5′-

TGCCACGCCCATCTTTAT-3′

***S100A9*** (f) 5′-TCAAAGAGCTGGTGCGAAA-3′ (r) 5′-

CAGCTGCTTGTCTGCATTTG-3′

***Tenascin C*** (f) 5′-CTTTGGCTGGGTTGCTTGAC-3′ (r) 5′-GTGCCAGGAGACCGTACCAC-3′

**Decorin** (f) 5′-CGCCTCATCTGGAGGGAGCTT-3′ (r) 5′-CTTTGGCTGGGTTGCTTGAC-3′

**TIMP1** (f) 5′-GTAGACGAACCGGATGTCAG -3′ (r) 5′- GAAGTCAACACCTTAT-3′

**TIMP2** (f) 5′-AGGGCCTGAGAATATAGAG-3′ (r) 5′-GGCCTTTCCAATGAGARA-3′

**MMP3** (f) 5′-ACCCACCTTACATACAGGATT-3′ (r) 5′-ACCCACCTTACATACAGGATTG-3′

### Measurement of cytokine release by ELISA

Cell culture supernatants were collected from tenocytes stimulated with 1 µg/ml S100A8 & S100A9, 10 ng/ml IL-1ß for 24 hours and the concentrations of IL-6, IL-8, CCL2 (Thermo Fisher Scientific, range 2000-31.25 pg/ml), CCL20, CXCL10 (Biolegend, CCL20 range 160-5 pg/ml, CXCL10 1000-15.6 pg/ml)), S100A8 and S100A9 (R & D systems, range 2000-31.25 pg/ml) were determined using commercially available ELISA kits. Cell culture supernatants were diluted with assay diluent to achieve concentrations within the specified range. Optical density was measured at 450 nm by a microplate reader.

### Histology and immunohistochemistry techniques

Samples were stained with H&E and toluidine blue for determination of the degree of tendinopathy as assessed by a modified version of the Bonar score (Grade 4 = marked tendinopathy, Grade 3 = advanced tendinopathy, Grade 2 = moderate degeneration, Grade 1 = mild degeneration, Grade 0 = normal tendon). This included the presence or absence of oedema and degeneration together with the degree of fibroblast cellularity and chondroid metaplasia. Thereafter, sections were stained with a range of primary antibodies directed against the following markers: S100A8 monoclonal antibody (Abnova MAB7961 Clone CF-145, UK; 2 µg/mL, 1:500 dilution), and S100A9 polyclonal antibody (Abnova PAB11470, UK; 2 µg/mL, 1:250 dilution).

Endogenous peroxidase activity was quenched with 3% (v/v) H_2_O_2_, and non-specific antibody binding blocked with 2.5% horse serum in Tris buffered saline (TBS) solution with detergent Tween20 (TBST) buffer for 30 min. Antigen retrieval was performed in 0.01 M citrate buffer for 20 min in a microwave. Sections were incubated with primary antibody in 2.5% (w/v) horse serum/human serum/TBST at 4 °C overnight. After two washes, slides were incubated with Vector ImmPRESS Reagent kit as per manufacturer’s instructions for 30 min. The slides were washed and incubated with Vector ImmPACT DAB chromagen solution for 2 minutes followed by extensive washing. Finally, the sections were counterstained with haematoxylin.

Images were captured using Apple Open laboratory software. Positive (human tonsil tissue) control specimens were included, in addition to the surgical specimens for each individual antibody staining technique and double immunofluorescence staining. Omission of primary antibody and use of negative control (human OA samples) isotypes confirmed the specificity of staining.

We applied a scoring system based on previous methods to quantify the immunohistochemical staining. Five random high-power fields (×400) were evaluated by two independent assessors (NLM, JHR). In each field, the number of positive and negatively stained cells were counted and the percentage of positive cells calculated giving the following semi quantitative grading: Grade 0 = no staining, Grade 1 = <10% cells stained positive, Grade 2 = 10–20% cells stained positive, Grade 3 = >20% cells positive.

### Protease Array

Cell supernatants were evaluated for the presence and relative amounts of 35 proteases using the Proteome Profiler Human Protease Array Kit (R&D Systems) according to the manufacturer’s protocol. Equal volumes of supernatants from tenocytes stimulated 1 µg/ml recombinant human S100A8 (Abcam), S100A9 (Abcam), 10 ng/ml IL-1ß for 24 hours were pooled from three donors and applied to the respective array membrane. Samples were then mixed with a combination of biotinylated detection antibodies and incubated overnight at 4 °C. The membranes were then subject to a series of washes before addition of diluted solution of horseradish peroxidase-conjugated streptavidin at room temperature for 30 minutes. Visualization of protease expression was carried out by chemiluminescence and signal intensity was quantified using an Azure c500 imaging system (Azure Biosystems). Relative optical densities of immunoreactive bands were determined using Image Studio Lite software (Li-Cor Biosciences).

### Statistical analysis

All values are expressed as mean ± SEM. Statistical analysis was performed using Kruskal-Wallis one-way analysis of variance or Student’s *t*-test. Values of *p* = < 0.05 are considered statistically significant.

### Ethical approval information

All procedures and protocols were approved by the Ethics Committee under approval numbers Central Network, South East Health (HREC/96/55, HREC/14/130) and West of Scotland REC (REC14/WS/1035) with informed consent obtained and carried out in accordance with standard operative procedures.

## Supplementary information

Supplementary Figures

## Data Availability

L.A.N.C. has access to all the data and data are available upon request.
