# Supplementary material for: S100A8 & S100A9: Alarmin mediated inflammation in tendinopathy
Source: Sci Rep. 2019 Feb 6;9:1463. doi: 10.1038/s41598-018-37684-3 (PMC6365574; doi:10.1038/s41598-018-37684-3)
Supplement: Supplementary file 1 — Supplementary Figures [file 41598_2018_37684_MOESM1_ESM.pdf]

S100A8 & S100A9: Alarmin mediated inflammation in tendinopathy

Lindsay AN Crowe[1], Michael McLean[1], Susan M Kitson[1], Emma Garcia Melchor[1], Katharina Patomell[1], Hai Man Cao[3], James H Reilly[1], William J Leach[2], Brain P Rooney[2], Simon J Spencer[2], Michael Mullen[2], Max Chambers[2], George AC Murrell[3], Iain B McInnes[1], Moeed Akbar[1] and Neal L Millar[1].

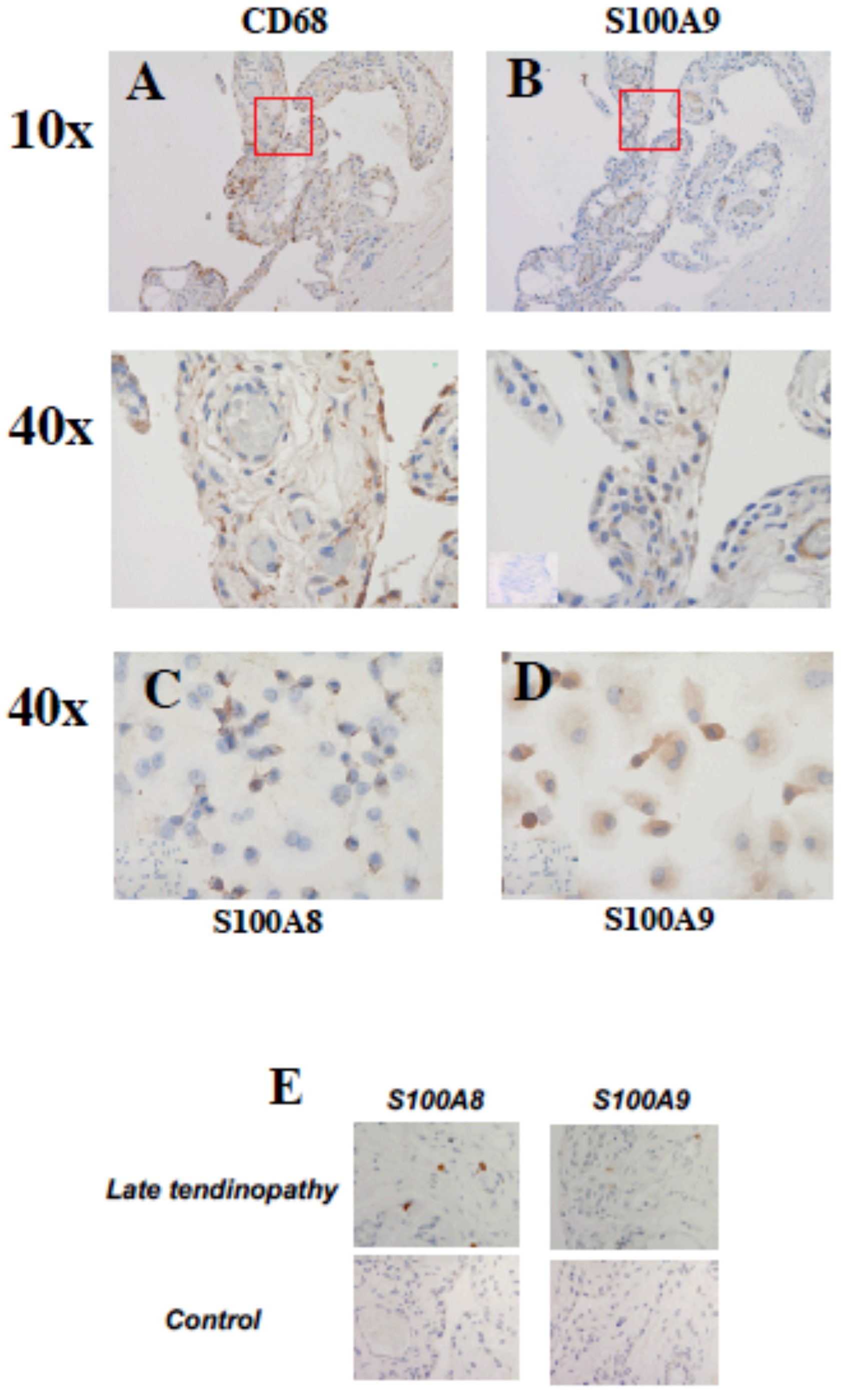

Supplementary Fig. S1

(A) (B) shows positive staining for CD68 (pan macrophage marker) localised to positive staining of S100A9. **(C&D)** Macrophages showing positive staining for S100A8 and S100A9**. (E)** Immunostaining of S100A8 and S100A9 in late tendinopathy (supraspinatus biopsy) and control (subscapularis) tendinopathy.

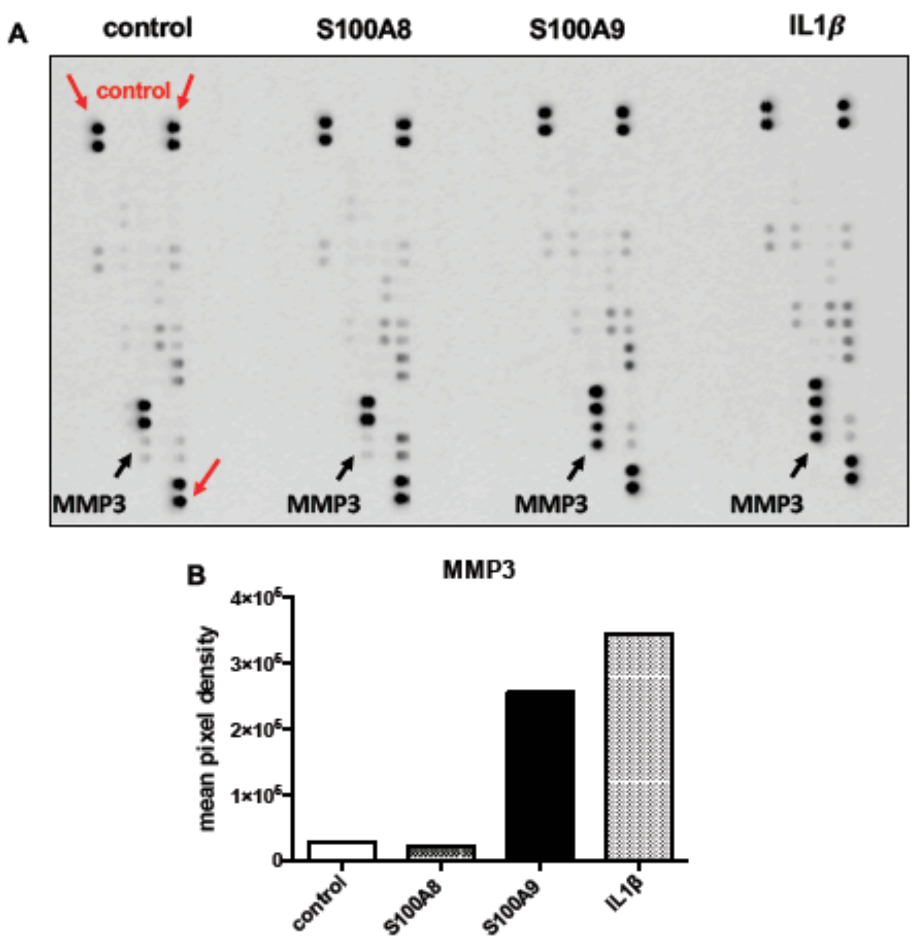

Supplementary Fig. S2

(A) Single sample protease array using supernatants from tenocytes stimulated with 1ug/ml S100A8, 1ug/ml S100A9 or 10ng/ml IL-1β for 24 hours with unstimulated control. (B) Signal pixel density of MMP3 plotted relative to control. Statistical analysis was not performed.

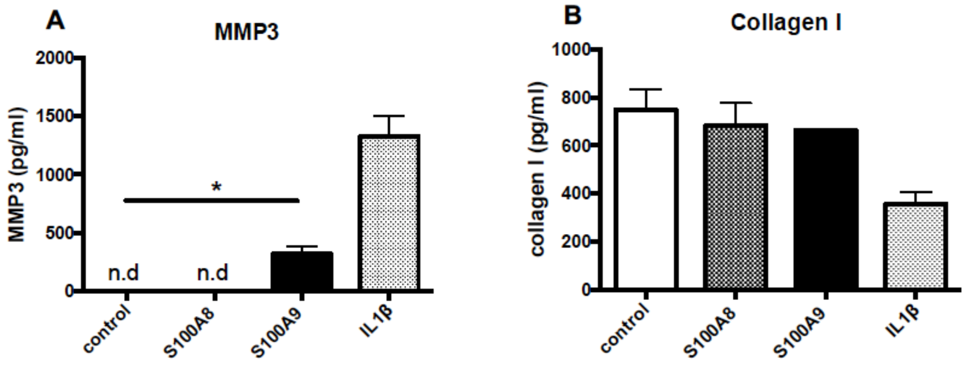

Supplementary Fig. S3

Concentration of MMP3 (A) and collagen I (B) in cell culture supernatants (expressed as pg/ml) from tenocytes stimulated with 1ug/ml S100A8, 1ug/ml S100A9 or 10ng/ml IL-1$\boldsymbol{\beta}$ for 24 hours with unstimulated control. All data represent mean ± SEM, n=3, * $p<0.05$.
